# Genome-wide association study revealed some new candidate genes associated with flowering and maturity time of soybean in Central and West Siberian regions of Russia

**DOI:** 10.3389/fpls.2024.1463121

**Published:** 2024-10-11

**Authors:** Roman Perfil`ev, Andrey Shcherban, Dmitriy Potapov, Konstantin Maksimenko, Sergey Kiryukhin, Sergey Gurinovich, Veronika Panarina, Revmira Polyudina, Elena Salina

**Affiliations:** ^1^ Institute of Cytology and Genetics, Siberian Branch, Russian Academy of Sciences, Novosibirsk, Russia; ^2^ Kurchatov Center for Genome Research of ICG SB RAS, Novosibirsk, Russia; ^3^ Siberian Federal Scientific Centre of Agro-BioTechnologies RAS, Novosibirsk, Russia; ^4^ FSBSI Federal Scientific Center of Legumes and Groat Crops, Orel, Russia

**Keywords:** soybean, GWAS, flowering, TOE1, DELLA, SPL3, maturity

## Abstract

The duration of flowering and maturity is an important agricultural trait determining the suitability of a variety for cultivation in the target region. In the present study, we used genome-wide association analysis (GWAS) to search for loci associated with soybean flowering and maturity in the Central and West Siberian regions of Russia. A field experiment was conducted in 2021/2022 at two locations (Orel and Novosibirsk). A germplasm collection of 180 accessions was genotyped using SoySNP50K Illumina Infinium Bead-Chip. From the initial collection, we selected 129 unrelated accessions and conducted GWAS on this dataset using two multi-locus models: FarmCPU and BLINK. As a result, we identified 13 loci previously reported to be associated with duration of soybean development, and 17 new loci. 33 candidate genes were detected in these loci using analysis of co-expression, gene ontology, and literature data, with the best candidates being *Glyma.03G177500, Glyma.13G177400*, and *Glyma.06G213100*. These candidate genes code the Arabidopis orthologs *TOE1* (*TARGET OF EAT 1*), *SPL3* (*SQUAMOSA PROMOTER BINDING PROTEIN LIKE 3*), the DELLA protein, respectively. In these three genes, we found haplotypes which may be associated with the length of soybean flowering and maturity, providing soybean adaptation to a northern latitudes.

## 1 Introduction

Soybean is a short-day crop with a very strong response to photoperiod. In northern latitudes, under long-day (LD) conditions, it significantly delays flowering and maturity. Significant progress has recently been made in the genetics of soybean development. The main genes *E1*-*E4* (Liu et al., 2008; Watanabe et al., 2009; Watanabe et al., 2011; Xia et al., 2012) have been identified, which have the strongest effect on flowering and maturity time and provide adaptation to different latitudes (Kurasch et al., 2017; Liu et al., 2020; Zhang et al., 2021; Yao et al., 2024). Other genes that are significantly related to the duration of soybean development stages have been identified, including *E1Lb* (Zhu et al., 2019), *Tof5* (Dong et al., 2022), *Tof13* (Li H. et al., 2023), *Tof16* (Dong et al., 2021), *Tof18* (Kou et al., 2022), *Tof16* (Dong et al., 2021), *Tof18* (Kou et al., 2022), and *J* (Lu et al., 2017).

The search for new loci and potential candidate genes that control these traits in soybean continues. Only the GWAS Atlas database (https://bigd.big.ac.cn/gwas/) contains information on 696 associations between SNPs and “days to flowering trait” in soybean. In particular, Yang et al (Yang et al., 2022). found 92 SNPs associated with flowering time in response to active accumulated temperature in a wild soybean population. Among the proposed candidate genes, three (aspartic peptidase 1, serine/threonine-protein kinase, and SCAR2-like protein) showed differential expression patterns and had haplotypes associated with variations in flowering time. Wu et al. identified 101 QTNs and three candidate genes with non-synonymous substitutions (*GmHY5, GmPIF4c*, and *GmVRN1*) that are associated with flowering time responses to photo-thermal conditions in soybean (Wu et al., 2023).

Moreover, in addition to the search for new candidate genes and loci, work is underway functionally characterize previously identified ones. Bu et al. demonstrated that components of the evening complex, in particular the proteins LUX (LUX ARRHYTHMO) and ELF3 (EARLY FLOWERING 3), repress E1 under short-day conditions by binding to its promoter region (Bu et al., 2021). Additionally, Lin et al. showed that phytochromes A (E3 and E4) stabilize both LUX proteins and E1 itself, suggesting preventing them from degradation by the 26S proteasome (Lin et al., 2022). It was also demonstrated that *E1* regulates the major soybean florigens (*GmFT2a* (*FLOWERING LOCUS T*) and *GmFT5a*) through the *GmMDE* and *GmEID1* genes, respectively (Zhai et al., 2022; Qin et al., 2023). The current state of the genetics of soybean flowering is presented in many reviews (Cao et al., 2016; Lin et al., 2021; Luo et al., 2021; Zhang et al., 2022).

Most of the territory of the Russian Federation is not well suited for soybean cultivation, primarily due to the LD conditions during the period of soybean growth and development. In 2020, the majority of soybean planted area was concentrated in the Central and Far Eastern regions of Russia, 37.9% and 43.5%, respectively (Lukomets, 2021). To promote such a valuable crop to other regions of Russia, for example, to Syberia, photoperiod-insensitive cultivars are required. The aim of our work is to identify new loci that control the flowering and maturity time of soybean, under the conditions of the Central and West Siberian regions of Russia. The information obtained will facilitate soybean marker-assisted selection, helping to create cultivars that are most suitable for cultivation under these conditions.

## 2 Materials and methods

### 2.1 Plant materials

In total, we used 180 accessions: 169 accessions from the collection of the Siberian Federal Scientific Center of Agro-BioTechnologies of the Russian Academy of Sciences (SFSC RAS, Novosibirsk, Russia) and 11 soybean cultivars kindly provided by the Federal Scientific Center of Legumes and Groat Crops (FSC LGC, Orel, Russia). Information on the country and region of origin, and the type of breeding material (breeding line or cultivars) for accessions are presented in **Supplementary Table S1**.

### 2.2 Analysis of phenotypes

The field experiment in Novosibirsk (54°55′ N 82°59′) and Orel (53°03′ N 36°03′ E) in 2021 and 2022 to study the duration from emergence to flowering (DTF, days from emergence to flowering) and maturity (DTM, days from emergence to maturity) have been described previously (Perfil’ev et al., 2023). The observed phenotypes are presented in **Supplementary Table S1**.

As phenotypes for GWAS analysis, we used BLUP (Best Linear Unbiased Prediction) values. Using “H2cal” function in the “inti” package (Lozano-Isla, 2024), outliers were filtered, the broad-sense heritability of traits was assessed, and BLUP values were calculated for each region separately, using a formula where genotype and year were treated as random effects. Heritability was estimated using the method of Cullis et al (Cullis et al., 2006).

All available observations on the duration of BBD (Beginning bloom date) and MD (Pod maturity date) for Chinese accessions were obtained from the SoyOmics database (Liu et al., 2023) (https://ngdc.cncb.ac.cn/soyomics/index). For these observations, BLUP values were calculated using a formula in which genotypes, year, and location were treated as random effects.

### 2.3 Genotyping and quality control of SNPs and genotypes

In total, we have obtained 180 genotypes using the SoySNP50K Illumina Infinium BeadChip (Song Q. et al., 2013), including 175 genotyped previously (Potapova et al., 2023) and 5 additionally genotyped using the same chip, following the previously described protocol (Potapova et al., 2023). The raw data were processed with Genome Studio v2 (Illumina Inc., San Diego, CA, USA) and then converted to Plink format. The SNP positions in the genotype file were updated from Wm82.a1 to Wm82.a2, according to Song et al (Song et al., 2016). All SNPs and 180 genotypes were filtered in plink 2.0 (Chang et al., 2015) with the settings “–maf 0.05”, “–geno 0.1” and “–king-cutof 0.354”, resulting in 129 accessions and 30569 SNPs remaining. We additionally added 2 synthetic SNPs in the genotype file that correspond to functional alleles of the *E1* gene (T — *e1-nl* and *e1-fs*; С — *e1-as*; NA — *E1* and missing genotypes) and *E4* genes (C — *e4-SORE-1* and *e4-kes*; A — *E4*; NA — missing genotypes). Genotypes for the *E1* and *E4* genes have been published previously (Perfil’ev et al., 2023). These data were used for all subsequent analyses.

### 2.4 Population structure, kinship and phylogenetic analysis

PCA analysis and Kinship matrix VanRaden was performed in GAPIT (Wang and Zhang, 2021). The population structure was determined using ADMIXTURE (Alexander and Lange, 2011) with K values ranging from 1 to 15 in order to obtain the cross-validation error for each K and determine the most probable number of clusters with the minimal cross-validation error. We determined that accession belongs to a Q cluster if its membership coefficient > 50%; accessions that did not fall into any of the clusters were defined as having mixed ancestry. ADMIXTURE bar plot was visualized using pophelper 2.3.1 (Francis, 2017).

The Neighbor-Joining tree was built in TASSEL5 (Bradbury et al., 2007) and visualized using the ggtree package (Yu et al., 2017).

### 2.5 Genome-wide association study

GWAS was conducted using the GAPIT package (Wang and Zhang, 2021) in R using Fixed and random model Circulating Probability Unification (FarmCPU) (Liu et al., 2016) and Bayesian-information and Linkage-disequilibrium Iteratively Nested Keyway (BLINK) (Huang et al., 2019). First two PCA were used as covariates in both models. Bonferoni corection (α/N) was used as significant threshold where N is number of tested SNPs and α = 1. Manhattan plots and QQ plots were visualized using the CMplot package (Yin et al., 2021).

### 2.6 Candidate gene search

HaplotypeMiner was used to defined a LD block in which a significant SNP is located (Tardivel et al., 2019). We used r_vs_ measures of disequilibrium corrected by population structure (Q matrix from ADMIXTURE results) and kinship (Centered_IBS matrix from TASSEL5 (Bradbury et al., 2007)). HaplotypeMiner settings were as recommended in the original article (Tardivel et al., 2019), with the exception of the “max_marker_to_gene_distance” and “max_flanking_pair_distance values”, which we increased to 500 Kb. Paired r^2^ and D` values between SNPs were obtained using TASSEL5 (Bradbury et al., 2007).

The rule of QTL naming is as follows: q + trait (DTF, DTM or FM (flowering and maturity) when loci affects on both trait) + - + serial number, for example, (qDTF-1, q (QTL) DTF (days from emergence to flowering) 1 (serial number)). Found QTLs were compared to previously reported loci from SoyBase (Brown et al., 2021) (https://www.soybase.org/) and GWAS Atlas (https://ngdc.cncb.ac.cn/gwas/) (Tian et al., 2020).

The list of genes located within the established loci, based on the Wm82.a2 genome version, was obtained from SoyBase (https://www.soybase.org/dlpages/SeqScan/SeqScanInput.php). Subsequently, gene IDs were converted to names according to the Wm82.a4 genome version (https://www.soybase.org/correspondence/index.php).

To prioritize candidate genes, we examined which of them were coexpressed with a set of previously well-studied genes, regulating flowering and maturity of soybean (bait genes): *E1* (Xia et al., 2012), *E1Lb* (Xu et al., 2015; Zhu et al., 2019), *E1La* (Xu et al., 2015), *GmEID1* (Qin et al., 2023)*, GmFT2a*, *GmFT5a*, *Tof5* (Dong et al., 2022), *Tof18* (Kou et al., 2022), *Tof16* (Dong et al., 2021), *E2* (Watanabe et al., 2011)*, E3* (Watanabe et al., 2009)*, ELF3* (Lu et al., 2017), *GmLUX1* (Bu et al., 2021), and *GmLUX2* (Bu et al., 2021). For co-expression analysis, we used previously obtained data from an RNA-seq experiment (Wu et al., 2014). This experiment included 162 libraries obtained from leaves of six cultivars under three light conditions: SD, Short Day (SD) 10Light:14Dark; Long Day (LD) (16Light:8Dark), Shift, 3 weeks under LD and then plants were transferred to SD 5 days (Wu et al., 2014). Experiment metadata (BioProject: PRJNA219510) and TPM values (Transcripts Per Kilobase Million) were obtained from the Soybean Gene Expresion Atlas v2 (https://soyatlas.venanciogroup.uenf.br/) (Almeida-Silva et al., 2023). Main parameters for “Simple Tidy GeneCoEx”: 1) Gene selection was biased on variance of bait gene; 2) Edge selection: Pearson correlation coefficient values r ≥ 0.55 or r ≤ −0.55 and FDR-adjusted p-value ≤ 0.01; 3) Construction of the network object was done with resolution parameters set to 2.5.

Gene ontology was obtained using the “GO Term Enrichment Tool” from SoyBase. Gene descriptions were obtained from the Phytozome website (https://phytozome-next.jgi.doe.gov/). The search for orthologs in Arabidopsis genome was carried out using TAIR BLAST (https://v2.arabidopsis.org/Blast/).

### 2.7 Haplotype analysis of candidate genes

Haplotype information for candidate genes was obtained using the «HapSnap» module from the SoyOmics (https://ngdc.cncb.ac.cn/soyomics/) (Liu et al., 2023). HapSnap parameters: 1) Variation type without options «Synonymous» and «Unclassed»; 2) Variation quality control was the default setting, with the exception of Haplotype Count ≥ 20; 3) Accessions only from the improved cultivars group (for the genes in **Supplementary Table S5**, we additionally included accessions from the landrace and wild soybeans group). The association between haplotypes and BLUP_BBD and BLUP_MD was established using the R basic function «aov». Multiple comparison of haplotypes were conducted using the R function «TukeyHSD».

### 2.8 Development of DNA markers and PCR

DNA markers were developed using dCAPS Finder dCAPS Finder 2.0 (http://helix.wustl.edu/dcaps/) (Neff et al., 2002). The PCR mixture with a total volume of 25 µL contained 10 mM Tris-HCl, pH 8.5, 50 mM KCl, 0.1% Tween 20, 2 mM MgCl2, 0.25 mM of each primer, 50–100 ng of DNA, and 1 U Taq DNA polymerase (BiolabMix, Novosibirsk, Russia). PCR protocol: 5 min at 95°C; 12 cycles (95°C 15 s; 65°C, 10 s; 72°C, 10 s); 34 cycles (95°C 15 s; 60°C, 10 s; 72°C, 10 s); 1 min. at 72°C.

For dCAPS and CAPS markers, DNA restriction digestion was carried out in a reaction mixture of 20 µL, which included 8 µL of PCR products, 2 µL of 10× restriction buffer and 1 U restriction enzyme (SibEnzyme, Novosibirsk, Russia). The mixture was incubated overnight at the optimum temperature for each enzyme.

The PCR and restriction products were separated in a 2% agarose gel with ethidium bromide. The results of electrophoresis were visualized and photographed in UV using Gel Doc™ XR+ (Bio-Rad Laboratories, Inc., Hercules, CA, USA).

## 3 Results

### 3.1 Kinship analysis and population structure

The initial collection consists of 180 soybean accessions, including 144 from Russia, 35 from 12 different countries, and 1 of unknown origin. Among the Russian accessions, 98 are from Novosibirsk (including 4 cultivars and 94 breeding lines created at SFSC RAS), 25 are from the Amur region, and 21 are from 9 other Russian regions.

As a result of the kinship analysis, we discovered that our initial collection contains accessions with a very high degree of relatedness to each other (**Supplementary Figure S1A**) (Potapova et al., 2023). Because the statistical association between genotypes and phenotypes could be biased by cryptic relatedness (Yu et al., 2006; Hellwege et al., 2017; Tibbs Cortes et al., 2021), we decided to exclude highly related accessions based on the KING kinship coefficients. As a result, we used the remaining 129 accessions for further GWAS analysis (**Supplementary Figure S1B**).

**Figure 1:**
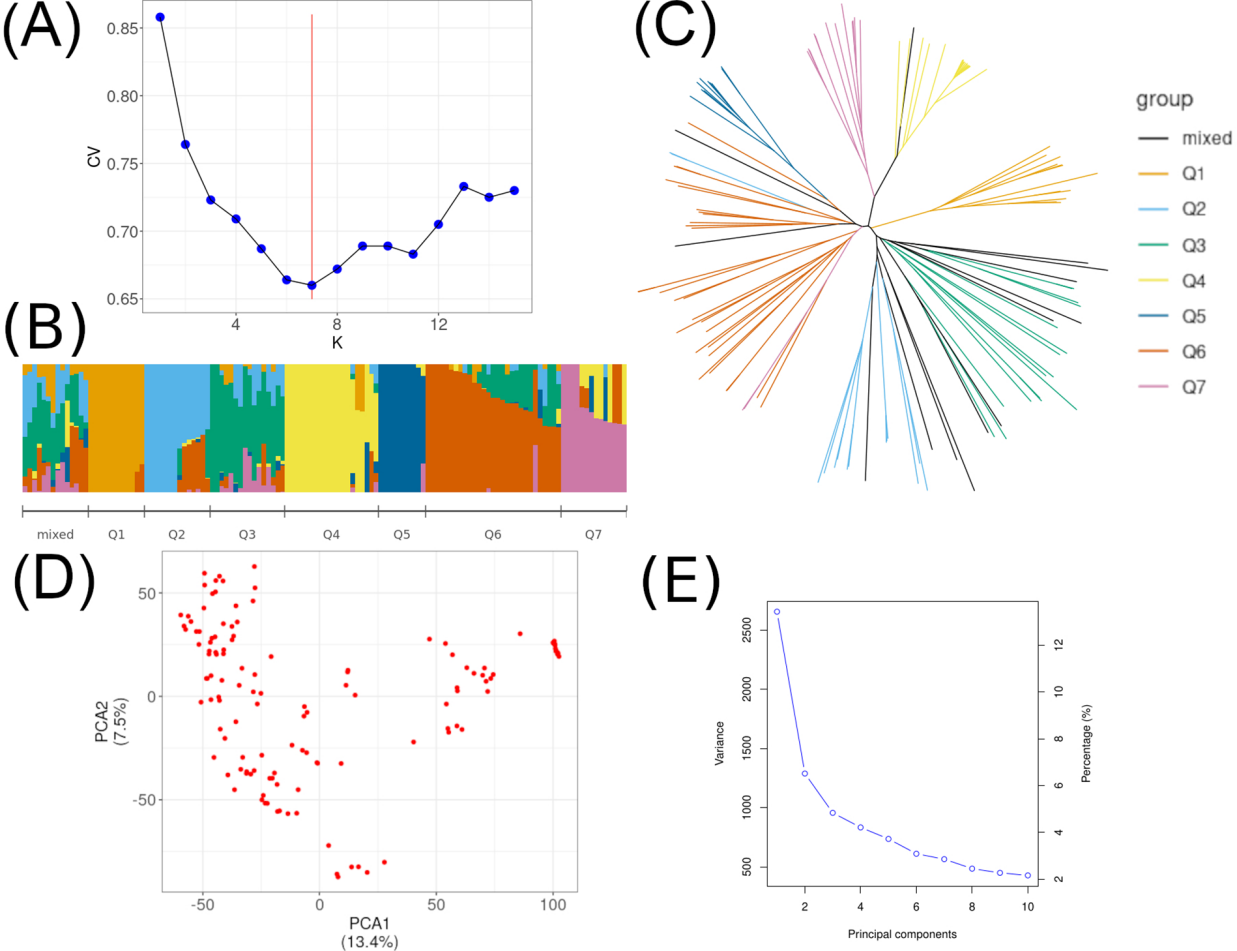
Population structure for 129 soybean accessions. **(A)** Cross-validation (CV) error for K from 1 to 15, where the red horizontal line denotes K = 7. **(B)** Population structure from the ADMIXTURE results for K = 7 and the mixed group. **(C)** A neighbor-joining tree of the 129 accessions that could be divided into 7 subpopulations and the mixed group. **(D)** The first two principal components (PCA) for 129 accessions. **(E)** Proportions of explained variance for the first 10 principal components.

Population structure and LD have previously been studied in the original set of accessions (Potapova et al., 2023). We re-examined the population structure using ADMIXTURE and PCA for 129 accessions in order to use the obtained Q-matrix in HaplotypeMiner, and the first two PCA as covariates in GWAS. Following the ADMIXTURE analysis, the lowest CV value (cross-validation) was identified at K = 7 (**Figures 1A, B**). Phylogenetic analysis also supports the clustering result (**Figure 1C**). The first two PCA are shown in the **Figures 1D, E**.

### 3.2 Phenotype analysis

**Figure 2:**
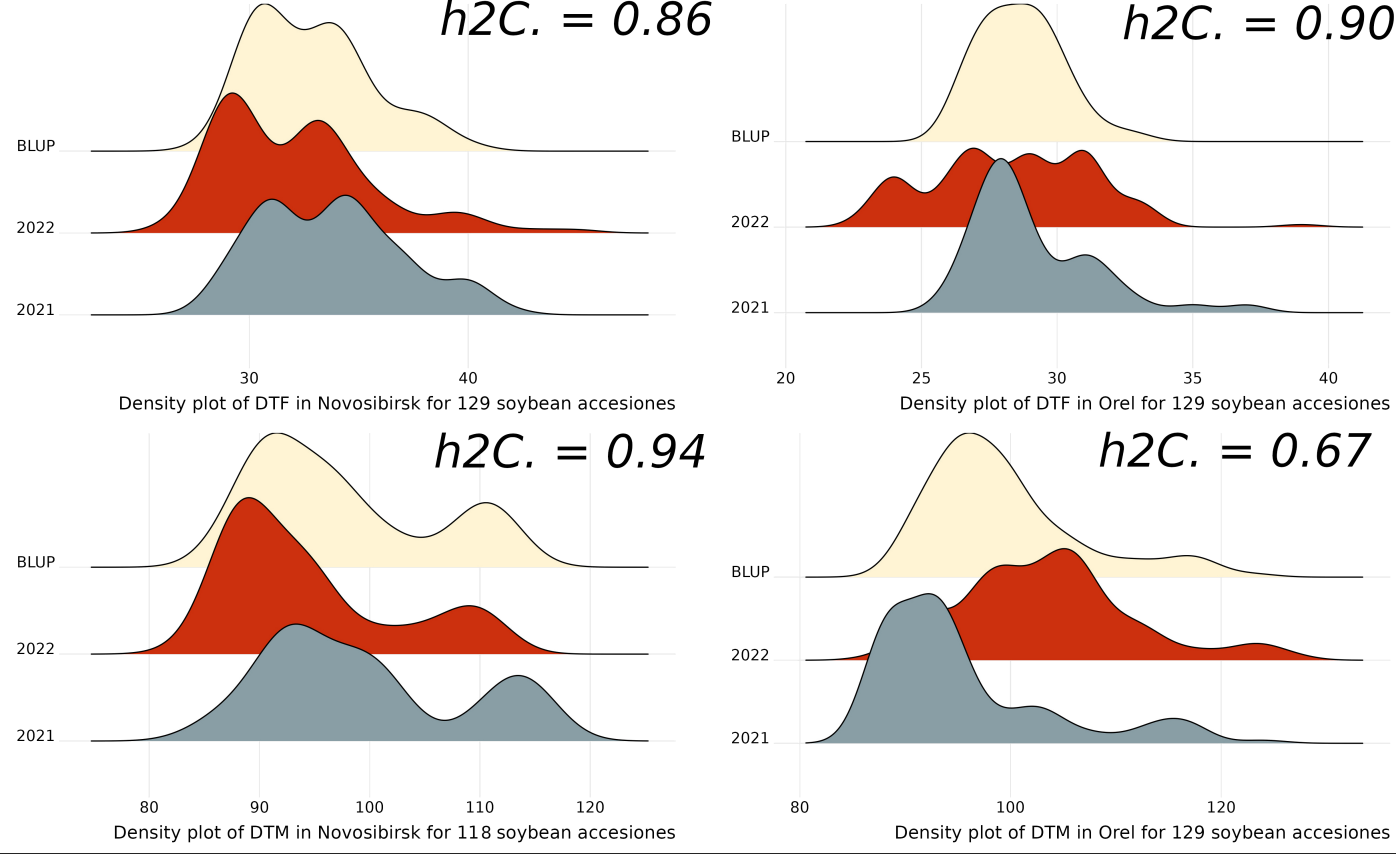
Density plots for DTF, DTM, and BLUP across years and regions studied, h2C indicates broad-sense heritability estimated using the Cullis method.

We used BLUP as phenotypes for GWAS analysis. The distribution of the obtained BLUP, DTF, and DTM by year in the studied regions, along with the heritability, is presented in **Figure 2**. The studied traits show high heritability values, among which the DTM value in Orel stands out. For the DTF trait, the BLUP values show a more normal distribution compared to the original observations, particularly for DTF in Orel. For the DTM trait, there is a bias towards earlier ripening, especially in Novosibirsk (**Figure 2**).

Descriptive statistics for phenotypes are presented in the **Supplementary Table S2**. On average, soybeans flowered earlier in Orel than in Novosibirsk, with the mean DTF being 29.1 and 28.3 in Orel, compared to 33.8 and 32 in Novosibirsk, in 2021 and 2022, respectively. In 2021, soybeans matured later in Novosibirsk than in Orel, with the mean DTM being 99.5 and 95.5, respectively. However, in 2022, the opposite situation occurred, namely, significantly later maturation in Orel compared with Novosibirsk, with the mean DTM being 103.4 and 94.4, respectively. We assume that this is due to the large amount of rainfall during the maturing of soybeans in 2022 in Orel (the sum of rainfall was ~250% of the average value for many years of observations) and the low temperatures during the first decade of September. We also believe that this has reduced the heritability value for DTM in Orel (**Figure 2**). In Orel, the maximum DTM values are higher than in Novosibirsk, which is due to the shorter growing season in Novosibirsk (**Supplementary Table S2**).

In the Novosibirsk region, among 129 accessions not all reached full maturity by the end of the growing season. Accessions that did not reach full maturity in at least one year were excluded from the BLUP calculation. As a result, 118 accessions remained for GWAS analysis for BLUP_DTM trait in the Novosibirsk region.

### 3.3 Association analysis

**Figure 3:**
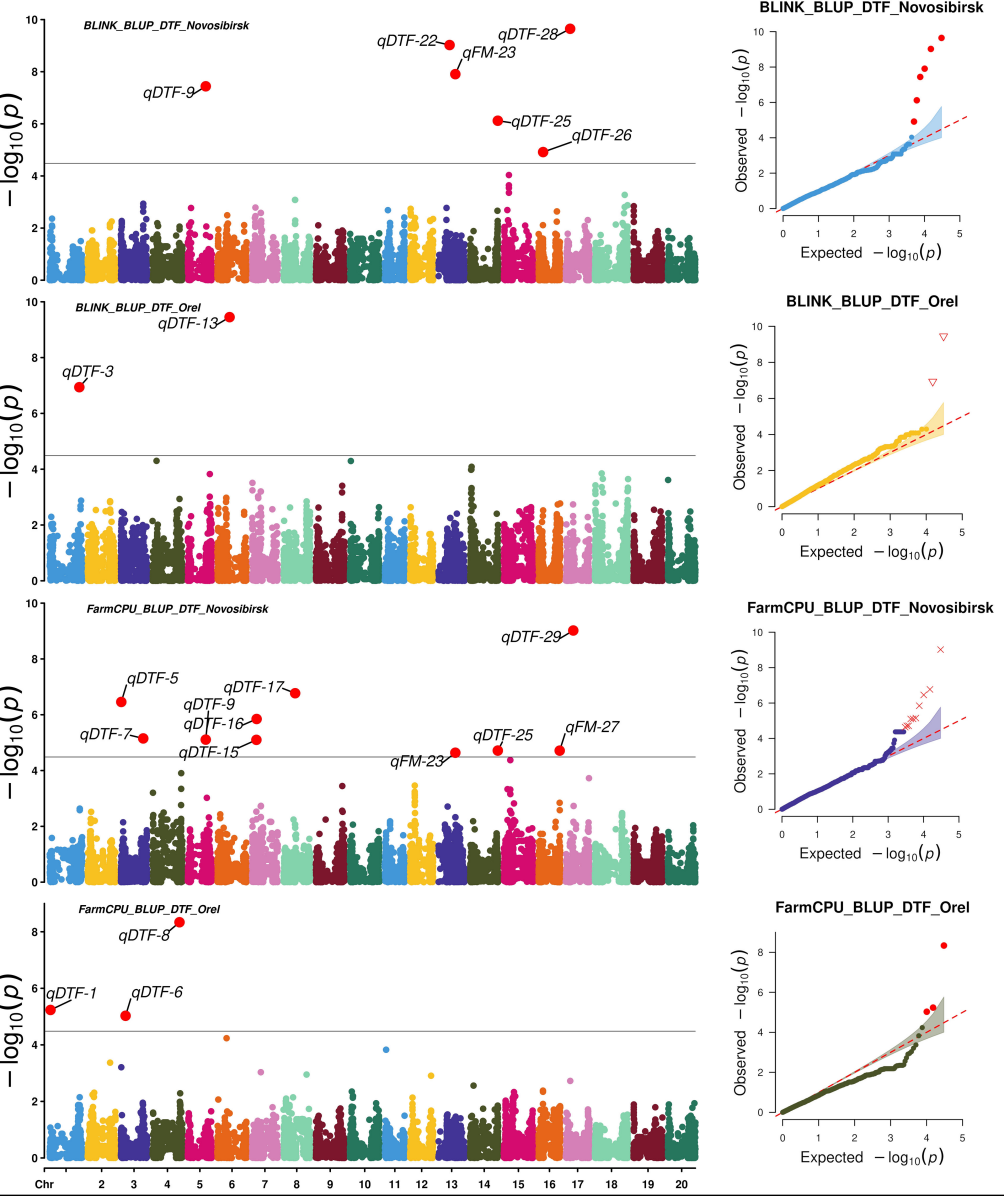
Manhattan plots and Q-Q plots for the associations obtained using FarmCPU and BLINK for BLUP_DTF in Orel and Novosibirsk.

**Figure 4:**
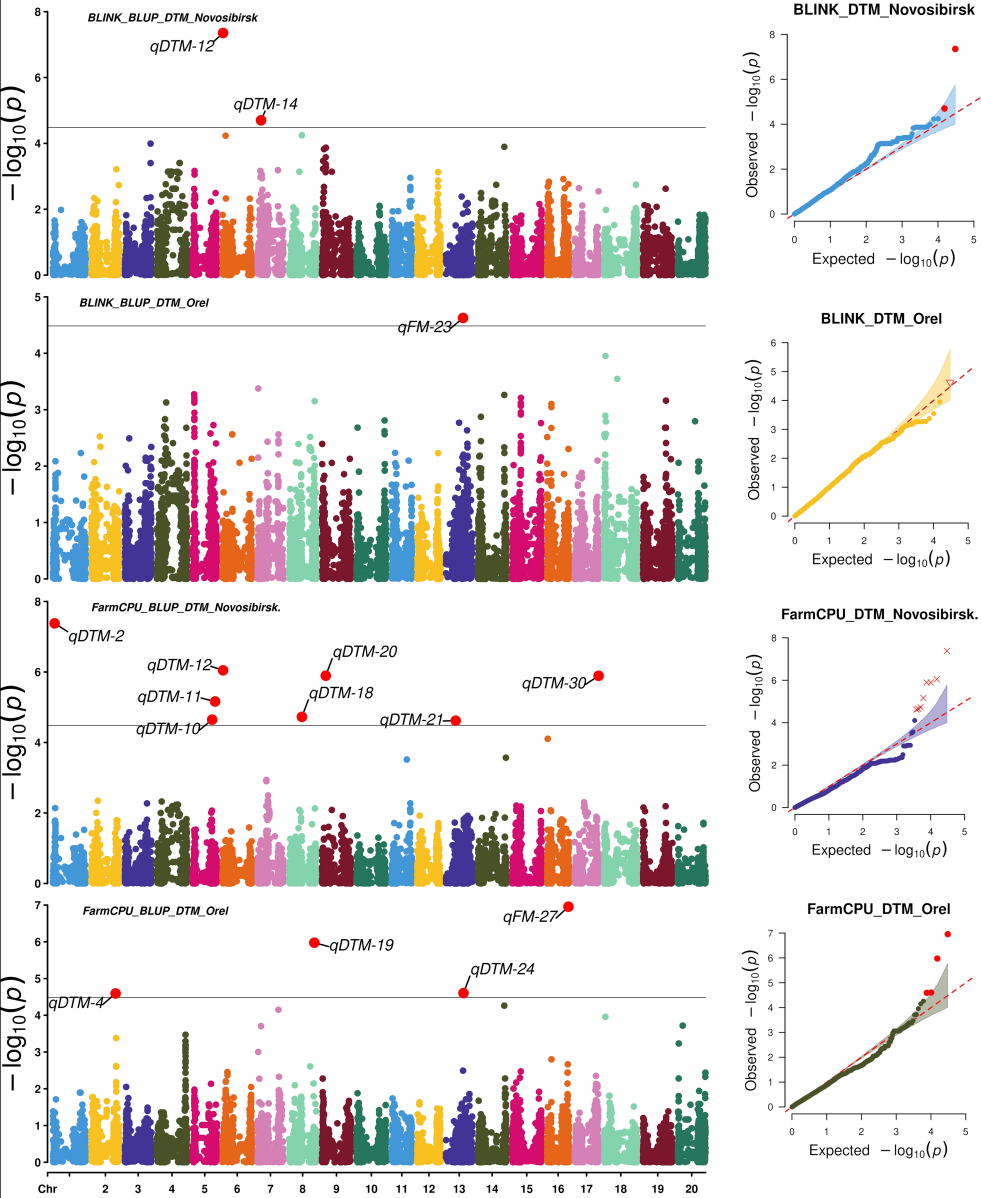
Manhattan plots and Q-Q plots for the associations obtained using FarmCPU and BLINK for BLUP_DTM in Orel and Novosibirsk.

To search for associations, we used two statistical models: FarmCPU and BLINK. FarmCPU and BLINK detected 13 and 9 SNPs associated with BLUP_DTF and BLUP_DTM in Novosibirsk, and 5 and 5 SNPs associated with BLUP_DTF and BLUP_DTM in Orel, respectively (**Figures 3**, **4**).

Using HaplotypeMiner, the LD blocks containing the significant SNPs were identified. For four SNPs, HaplotypeMiner could not determine the LD block for 4 SNPs (**Supplementary Table S3**). For these SNPs, we established that the locus size was within ±500 Kb, since the previously determined LD half-life for this dataset was ~1.2 Mb (Potapova et al., 2023). Based on the positions of SNPs and LD blocks in the genome, it was revealed that 32 significant SNPs correspond to 30 loci. In total, 13 and 5 loci associated with BLUP_DTF were identified under the conditions of Novosibirsk and Orel, respectively (**Table 1**, **Figures 3**). For BLUP_DTM, 9 and 5 loci were identified under the conditions of Novosibirsk and Orel, respectively (**Figure 4**). All loci identified using FarmCPU and BLINK and their corresponding SNPs are presented in **Supplementary Table S3**. **Supplementary Figure S2** shows a Venn diagram with the intersection of established loci for the traits and regions studied. Two loci, qFM-23 and qFM-27, are associated with BLUP_DTF in Novosibirsk and BLUP_DTM in Orel.

**Table 1:** Loci associated with BLUP_DTF and BLUP_DTM along with previously reported QTLs.

Loci	Region	High p-value SNPs	Chromosome	Position (Wm82,a2)	Effect	SoyBase and GWAS Atlas QTLs	References
qDTF-1	Orel	Gm01_1279146_C_T	1	1273914	-0.37	gmx24899 (days to flowering trait)	(Hu et al., 2020)
qDTM-2	Nov	Gm01_2809603_T_G	1	2825569	4.15	First flower 5-g18	(Fang et al., 2017)
qDTF-3	Orel	Gm01_50744456_A_C	1	51629974	0.52		
qDTM-4	Orel	Gm02_44923400_G_T	2	41847331	-2.16		
qDTF-5	Nov	Gm03_592600_T_C	3	591655	0.9		
qDTF-6	Orel	Gm03_8434431_A_G	3	8079197	-0.42		
qDTF-7	Nov	Gm03_40959110_A_G	3	38947582	-0.49	R8 full maturity 3-g4; gmx3002184 (days to maturity)	(Hu et al., 2014; Zimmer et al., 2021)
qDTF-8	Orel	Gm04_43966863_G_A	4	47114807	-0.5		
qDTF-9	Nov	Gm05_31055328_C_T	5	31321892	1.24		
qDTM-10	Nov	Gm05_33603547_T_C	5	33871249	-1.4		
qDTM-11	Nov	Gm05_41329275_G_A	5	39008584	-2.55	Gmx4880686 (days to maturity)	(Zimmer et al., 2021)
qDTM-12	Nov	Gm06_1197535_A_C	6	1215222	3.3		
qDTF-13	Orel	Gm06_21479787_C_T	6	21400435	0.73	gmx22972428 (days to flowering trait)	(Kim et al., 2020)
qDTM-14	Nov	Gm07_6015318_C_A	7	6053956	3.5	First flower 2-g6; First flower 7-g6	(Zhang J. et al., 2015)
qDTF-15	Nov	Gm07_7427187_C_T	7	7466583	-0.69	First flower 2-g7; First flower 7-g7	(Zhang J. et al., 2015)
qDTF-16	Nov	Gm07_7841877_G_A	7	7881251	-1.08		
qDTF-17	Nov	Gm08_21170988_C_T	8	21127737	-0.61		
qDTM-18	Nov	Gm08_22622648_A_G	8	22578609	-2.62		
qDTM-19	Orel	Gm08_43298914_A_C	8	44133893	-3.1		
qDTM-20	Nov	Gm09_6574547_A_G	9	6532643	-2.16	gmx7966749 (days to maturity); gmx22972958 (days to full bloom)	(Li et al., 2019; Zimmer et al., 2021)
qDTM-21	Nov	Gm13_5035568_C_T	13	16796464	-3.3	gmx11563119 (days to seed formation)	(Yan et al., 2021)
qDTF-22	Nov	Gm13_2584486_G_A	13	19282735	0.8	gmx11604810 (days to seed formation)	(Yan et al., 2021)
qFM-23	Nov	Gm13_28141969_T_G	13	29335356	0.73	Reproductive stage length 4-g3	(Copley et al., 2018)
	Orel	Gm13_28307256_G_A	13	29500887	3.3		
qDTM-24	Orel	Gm13_29071734_A_G	13	30271391	1.54	gmx11818420	(Li et al., 2019)
qDTF-25	Nov	Gm14_49487340_C_T	14	48281947	0.6		
qDTF-26	Nov	Gm16_7670126_A_C	16	7828040	0.7		
qFM-27	Nov	Gm16_36274380_T_C	16	36777185	0.73	gmx15489199 (days to maturity); gmx15507322 (days to maturity)	(Zimmer et al., 2021)
	Orel	Gm16_36274380_T_C	16	36777185	2.8		
qDTF-28	Nov	Gm17_8014179_C_T	17	7744734	-0.8	gmx15646668; (days to maturity) gmx22974074 (days to maturity)	(Ravelombola et al., 2021; Zimmer et al., 2021)
qDTF-29	Nov	Gm17_13487812_A_G	17	13225475	0.71		
qDTM-30	Nov	Gm17_41866889_G_A	17	41600558	2.4		

### 3.4 Co-localization with previously identified QTLs and prioritization of candidate genes

To search for previously established QTLs located within the LD blocks, we used data from GWAS Atlas (https://ngdc.cncb.ac.cn/gwas/) and SoyBase. We found that 13 of the 30 loci co-localize with previously identified QTLs that are associated with different phases of soybean developmental (**Table 1**).

Within the 30 identified loci, there are 1269 genes. To prioritize and select the most interesting candidate genes, we studied which of them are co-expressed with a set of 14 bait genes (previously well-studied genes) involved in soybean flowering and maturity using the “Simple Tidy GeneCoEx” algorithm (Li C. et al., 2023).

For constructing the gene network, we used data from the RNA-seq experiment by Wu et al (Wu et al., 2014), which examined the response of soybean leaves to photoperiod. We believe that the residual reaction of soybean to photoperiod, under our conditions, is one of the main factors influencing the growth and development of soybean.

Out of 1269 genes, we selected 1022 genes to calculate the correlation coefficient. These selected genes have higher expression variation than the *Tof18*/*Glyma.18G224500* bait gene, which has the lowest variation compared to other bait genes (**Supplementary Figure S3A**). To construct the graph, we used a resolution parameter of 2.5, which we chose as a compromise value between the two performance indexes (**Supplementary Figure S3B**). Thus, the constructed graph consists of 816 genes and 17 modules containing more than 5 genes (**Supplementary Figure S3B**).

In the resulting gene network, 546 genes are co-expressed with the selected set of bait genes. These co-expressed genes were studied in more detail, based on their GO and literature data. Among the GO terms, we first looked at the biological function, namely: participation of genes in the response to hormones, in the processes of growth and development and in the response to light signals. As a result, 33 candidate genes were identified as the most promising for further study. **Supplementary Table S4** presents the identified candidate genes and the bait genes with which they are significantly co-expressed. For 8 loci, we were unable to propose candidate genes.

### 3.5 Analysis of haplotypes of candidate genes and their distribution in different regions of China and major soybean populations

**Figure 5:**
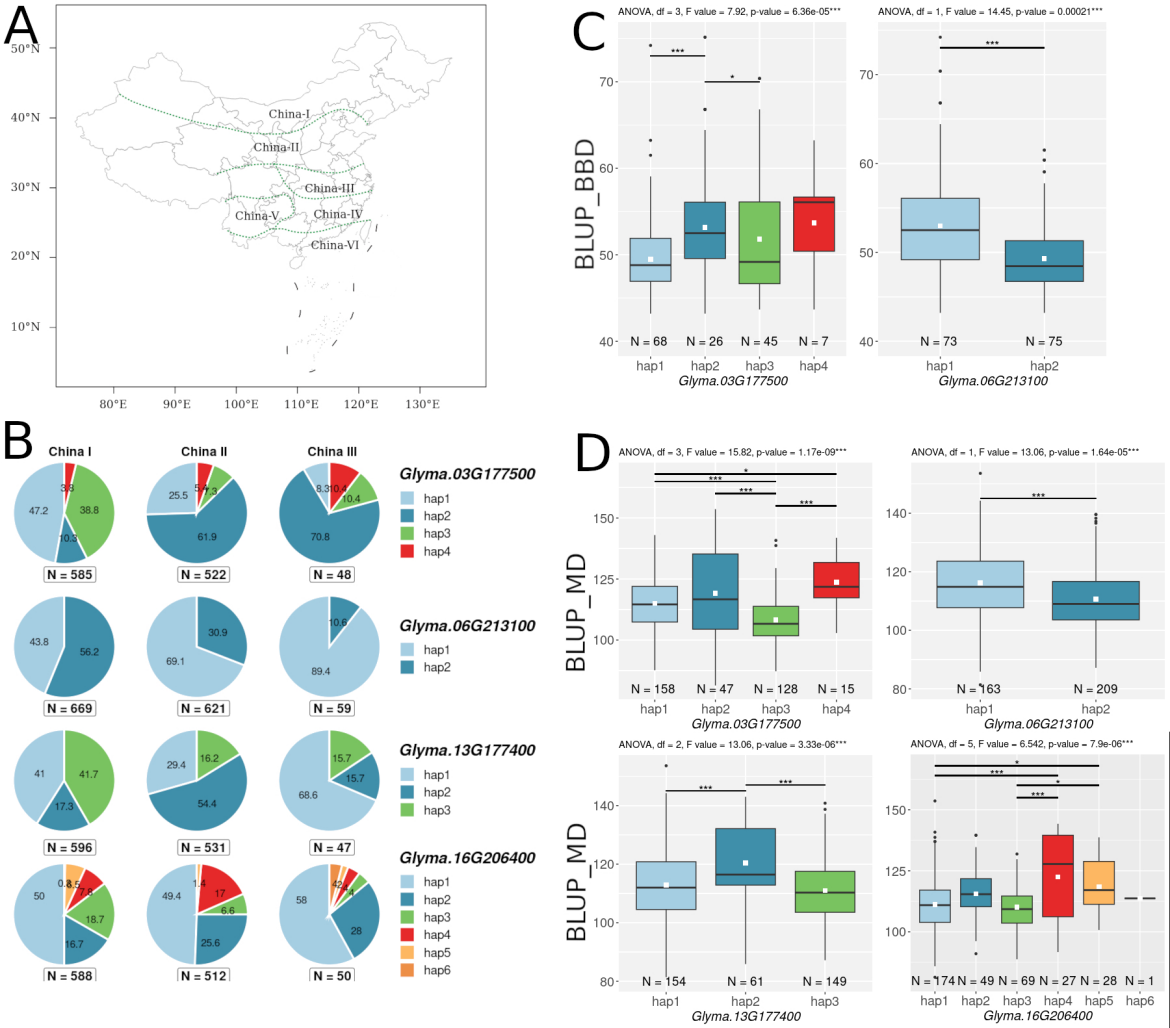
**(A)** Map of China divided into 6 regions, obtained from the SoyOmics website (https://ngdc.cncb.ac.cn/soyomics/index). **(B)** Distribution of haplotypes of 4 candidate genes in the 3 regions of China. Numbers on pie charts indicate percentages. “N” denotes the number of improved cultivars. **(C)** Association of haplotypes *Glyma.03G177500* and *Glyma.06G213100 with* BLUP_BBD. **(D)** Association of haplotypes *Glyma.03G177500*, *Glyma.06G213100*, *Glyma.13G177400*, and *Glyma.16G206400* with BLUP_MD. White squares on the box plots indicate the mean value. Asterisks indicate significant differences between the compared haplotypes: ***p < 0.001; *p < 0.05.

Using the SoyOmics database, we studied the presence of functional variation (substitutions that affect the protein structure) in the coding regions of 33 candidate genes in improved soybean cultivars. Additionally, we analyzed the association of their haplotypes with BLUP_BBD (Beginning Bloom Date) and BLUP_MD (Pod Maturity Date) in soybean cultivars from the northern region of China (China I region in SoyOmics, **Figure 5A**). Two genes, *Glyma.03G006600* and *Glyma.08G320700*, could not be analyzed because they are not represented in the ZH13.a2 genome assembly. Ten genes do not contain any functional nucleotide substitutions; one gene, *Glyma.16G076600*, contains a mutation leading to a frame shift, and the remaining genes contain various non-synonymous mutations (**Supplementary Table S4**).

As a result of association analysis, two genes (*Glyma.03G177500* and *Glyma.06G213100*) were found to be associated (p-value < 0.001) with BLUP_BBD (**Figure 5C**), and four genes (*Glyma.03G177500*, *Glyma.06G213100*, *Glyma.13G177400*, and *Glyma.16G206400*) were found to be associated with BLUP_MD (**Figure 5D**). Additionally, for these four genes, we examined the distribution of their haplotypes in cultivars from three soybean growing regions of China (China I, China II, and China III regions in SoyOmics, **Figures 5A, B**). **Supplementary Table S5** presents the identified haplotypes and the substitutions forming them for four genes: *Glyma.03G177500*, *Glyma.06G213100*, *Glyma.13G177400*, and *Glyma.16G206400.* Additionally, we examined the variation in haplotype frequencies for these four genes across three major soybean populations: wild soybeans (*Glycine soja* Sieb. & Zucc.), landraces, and improved cultivars (**Supplementary Table S5**). The *Glyma.03G177500*, *Glyma.13G177400*, and *Glyma.16G206400* genes show a bias in haplotypes frequency from wild toward cultivated soybeans (**Supplementary Table S5**).

### 3.6 Analysis of haplotypes *Glyma.03G177500*, *Glyma.06G213100* and *Glyma.13G177400* in the studied collection

We attempted to analyze the haplotypes of three candidate genes (*Glyma.03G177500*, *Glyma.06G213100*, and *Glyma.13G177400*) using DNA markers. These genes are associated with BLUP_BBD and BLUP_MD (**Figures 5C, D**), and their haplotype frequencies change with the spread of cultivars from north to south in China (**Figures 5A, B**).

**Figure 6:**
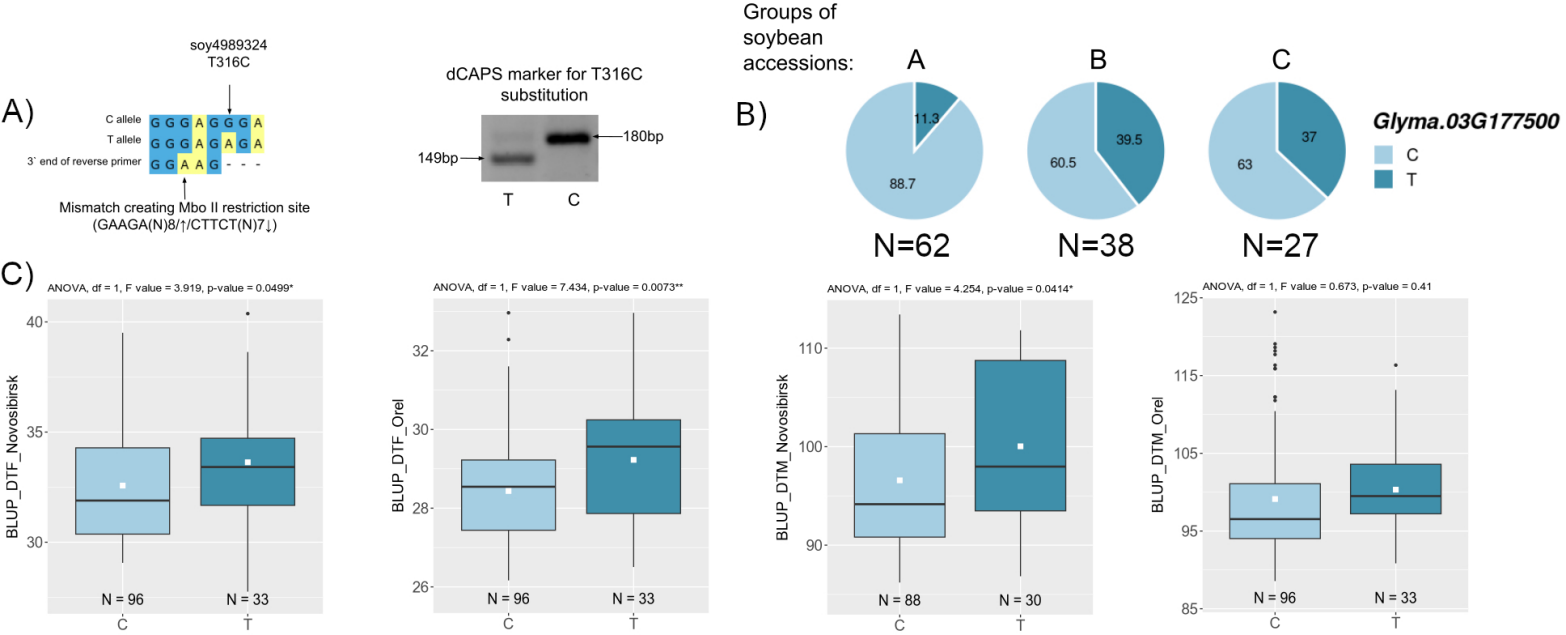
**(A)** Design and electrophoresis of the dCAPS marker for genotyping the soy4989324 mutation. **(B)** Distribution of soy4989324 in three groups of soybean accessions: A—from West Siberia (Novosibirsk and Omsk regions); B—from other Russian regions; and C—from other countries. Numbers on pie charts indicate percentages. “N” denotes the number of accessions. **(C)** Association of soy4989324 mutation with BLUP_DTF and BLUP_DTM in Orel and Novosibirsk. White squares on the box plots indicate the mean value. Asterisks indicate significant differences between the compared allele: **p < 0.01; *p < 0.05.

**Figure 7:**
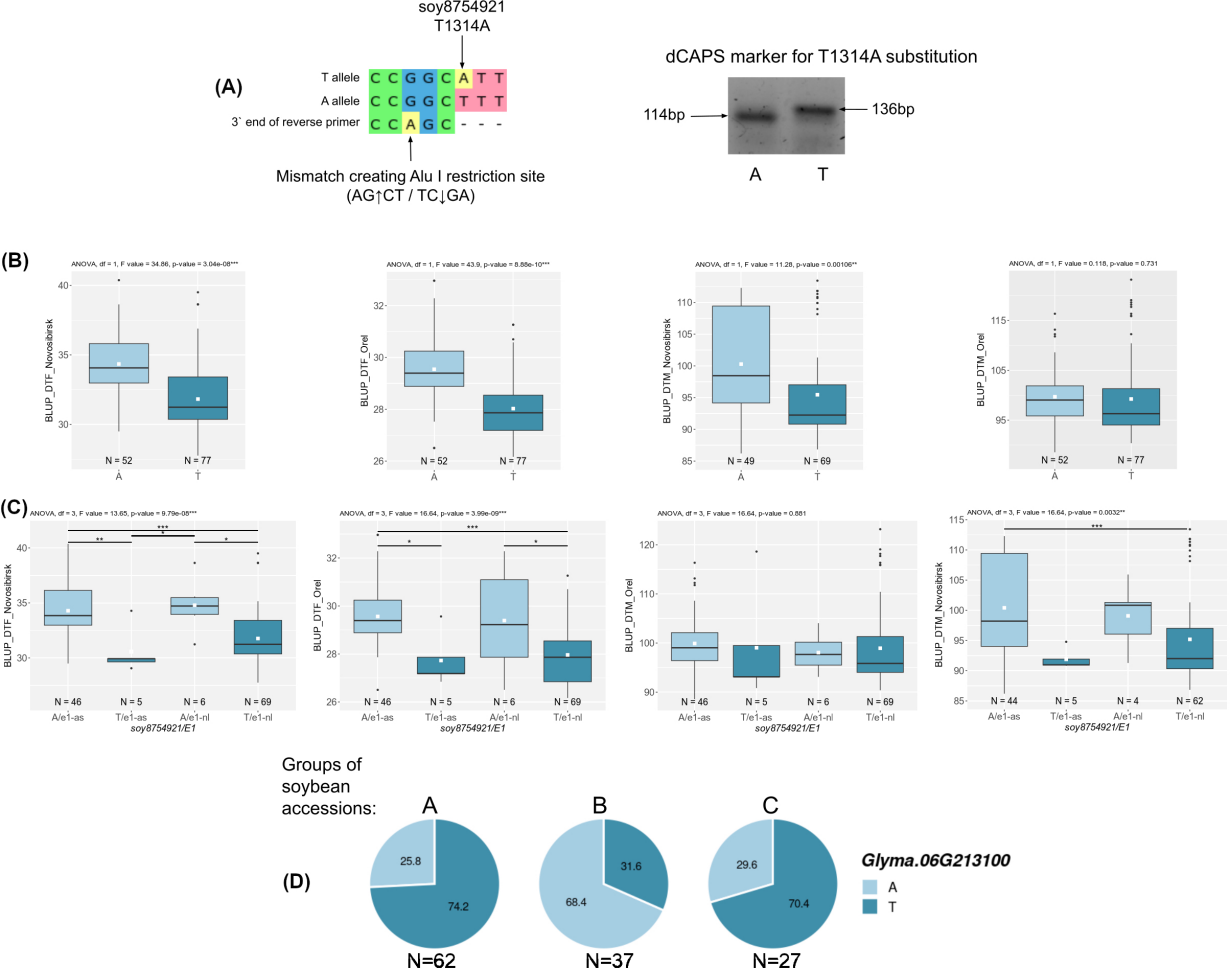
**(A)** Design and electrophoresis of the dCAPS marker for genotyping the soy8754921 mutation. **(B)** Association of soy8754921 mutation with BLUP_DTF and BLUP_DTM in Orel and Novosibirsk. **(C)** Association of soy8754921/*E1* genotype with BLUP_DTF and BLUP_DTM in Orel and Novosibirsk. White squares on the box plots indicate the mean value. Asterisks indicate significant differences between the compared genotype: ***p < 0.001; **p < 0.01; *p < 0.05. **(D)** Distribution of soy8754921 in three groups of soybean accessions: A—from West Siberia (Novosibirsk and Omsk regions); B—from other Russian regions; and C—from other countries. Numbers on pie charts indicate percentages. “N” denotes the number of accessions.

**Figure 8:**
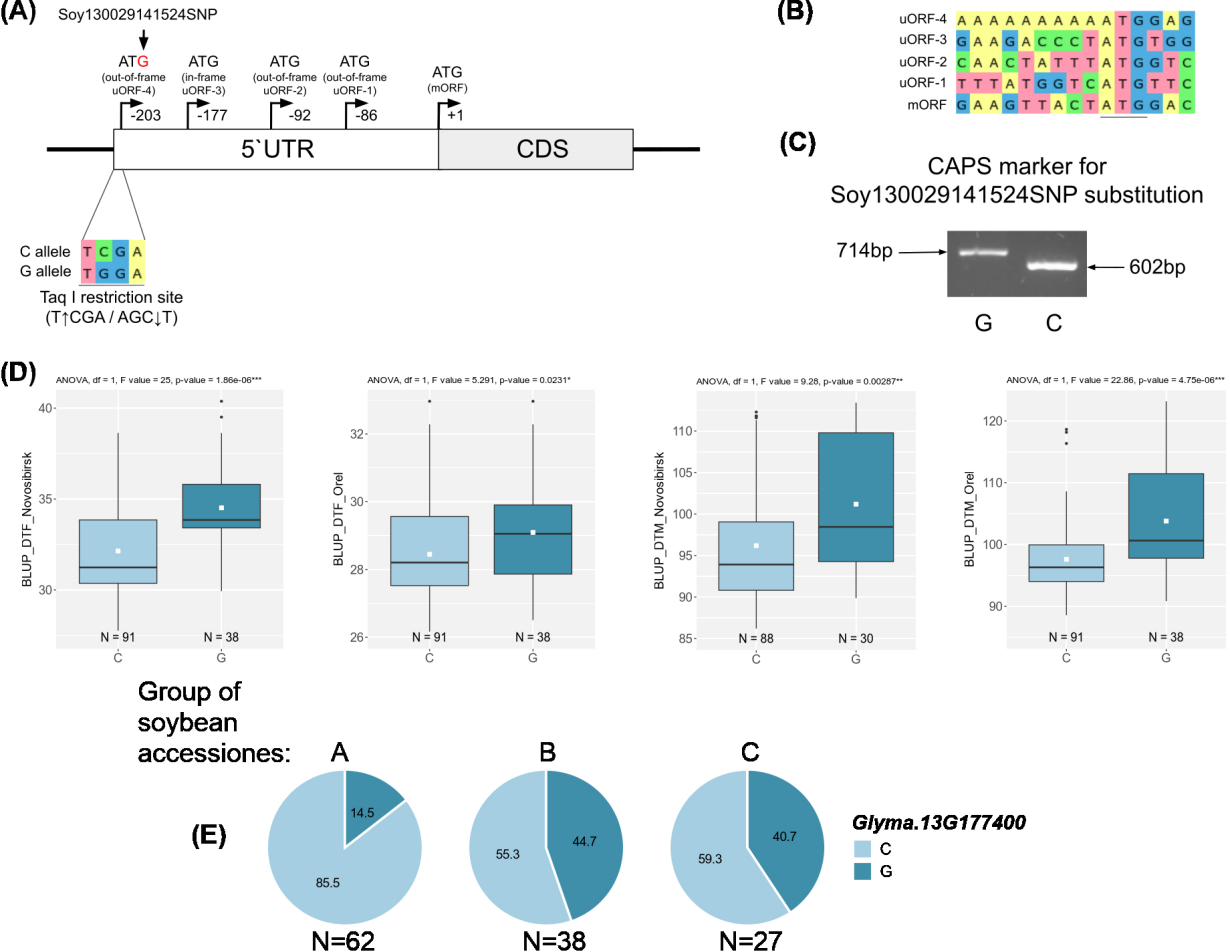
**(A)** Design of the CAPS marker for genotyping the Soy130029141524SNP mutation. Upstream open reading frames (uORFs) in 5`UTR of the *Glyma,13G177400*. **(B)** Sequences around ATG codons in uORFs and mORF. **(C)** Electrophoresis of the CAPS marker for genotyping of the Soy130029141524SNP mutation. **(D)** Association of Soy130029141524SNP mutation with BLUP_DTF and BLUP_DTM in Orel and Novosibirsk. White squares on the box plots indicate the mean value. Asterisks indicate significant differences between the compared allele: ***p < 0.001; **p < 0.01; *p < 0.05. **(E)** Distribution of Soy130029141524SNP in three groups of soybean accessions: A—from West Siberia (Novosibirsk and Omsk regions); B—from other Russian regions; and C—from other countries. Numbers on pie charts indicate percentages. “N” denotes the number of accessions.

For two candidate genes *Glyma.03G177500* and *Glyma.06G213100*, we successfully developed dCAPS markers for two non-synonymous nucleotide substitutions soy4989324 and soy8754921 (**Figure 6A**, **7A**). The first mutation, soy4989324, distinguishes the early flowering and maturing haplotypes, hap1 and hap3, from the late haplotypes, hap2 and hap4 (**Figures 5C, D**, **Supplementary Table S5**). The second mutation, soy8754921, is the only nucleotide substitution detected in the *Glyma.06G213100* gene (**Supplementary Table S5**). The *Glyma.13G177400* gene could not be analyzed in a similar way; the developed DNA markers did not detect it’s polymorphism. We additionally studied the natural variation of *Glyma.13G177400* using the SoyMD database (https://yanglab.hzau.edu.cn/SoyMD/#/) (Yang et al., 2024) and found a Soy130029141524SNP mutation, which creates an alternative start codon in the in 5`UTR. To study this polymorphism, we developed a CAPS marker (**Figure 8C**, **Supplementary Table S6**). Using the developed DNA markers, we genotyped 129 accessions that were used for GWAS analysis. The identified genotypes are presented in **Supplementary Table S1**. The obtained results allow to study the association between these SNPs and BLUP_DTF and BLUP_DTM in Orel and Novosibirsk (**Figures 6C**, **7B, C**, **8D**), and to examine the LD between high p-value SNPs from GWAS results and these mutations. Additionally, we studied the frequency of this substitutions in three groups of accessions: A—from West Siberia (Novosibirsk and Omsk regions); B—from other Russian regions; and C—from other countries. As a result, we found that alleles associated with early flowering and maturity (**Figures 6C**, **7B, C**, **8D**) are predominant in our collection, especially among accessions from group A (**Figures 6B**, **7D**, **8E**).

## 4 Discussion

Genome-wide association study (GWAS) is a powerful and ubiquitous tool for searching for new loci and genes that control various traits in plants. However, its deficiency and outcome are highly dependent on the diversity and size of the germplasm collection (Tibbs Cortes et al., 2021). One of the problems is the presence of duplicates among germplasm accessions that can result in spurious associations (Yu et al., 2006; Hellwege et al., 2017; Tibbs Cortes et al., 2021). Since our initial collection contains the large number of genetically similar accessions (**Supplementary Figure S1A**), we excluded them from analysis (**Supplementary Figure S1B**). Most of the excluded accessions are breeding lines created at the SFSC RAS (**Supplementary Table S1**). It is possible that such a decrease in genetic diversity in the collection is due to peculiarities in the breeding practices at the SFSC RAS. Only a small number of accessions can fully mature in Western Siberia. Therefore, the first Novosibirsk cultivar SibNIIK-315, well adapted to these conditions, was used in breeding most often. For example, the newest two Novosibirsk cultivars, SibNIIK-9 (Rozhanskaya and Polyudina, 2017) and Gorinskaya (Kashevarov et al., 2021), were obtained with the use of SibNIIK-315. Besides, in the breeding program of SFSC RAS, in addition to hybridization, the such methods as single plant selection from cultivars (Kashevarov et al., 2020), clonal selection (Rozhanskaya and Polyudina, 2017), and mutagenesis (Rozhanskaya and Polyudina, 2017) were used reducing the genetic diversity in the collection. The obtained genotyping results made it possible to reorganize and optimize the germplasm collection, in order to increase the efficiency of conservation and utilization of soybean genebank resources in this breeding center.

The found loci do not overlap with *E1* and *E4* genes, although they are present in this collection and also provide variation in DTF and DTM in the studied regions (Perfil’ev et al., 2023). A significant association of DTF and DTM with *E1* and *E4* (*E2* and *E3* were excluded due to low allele frequency) was obtained only using naive models without correction for population structure and kinship. It is possible that the allelic frequency of *E1* and *E4* is strongly correlated with the population structure or kinship in our collection, or there are some other factors influencing their association with BLUP_DTF and BLUP_DTM in the statistical models used for GWAS. However, 13 loci overlap with genomic regions previously reported to be associated with different phases of soybean development (Hu et al., 2014; Zhang J. et al., 2015; Fang et al., 2017; Copley et al., 2018; Li et al., 2019; Hu et al., 2020; Kim et al., 2020; Ravelombola et al., 2021; Yan et al., 2021; Zimmer et al., 2021), which adds confidence to the established associations (**Table 1**).

Co-expression analysis, as part of a post-GWAS analysis, has previously been used to search for candidate genes associated with resistance to fungal pathogens (Almeida-Silva and Venancio, 2021), pests (Almeida-Silva and Venancio, 2023), unsaturated fatty acid content (Zhao et al., 2024a) and oil content (Zhao et al., 2024b) in soybean. In this work, this approach has been applied to prioritize candidate genes associated with flowering and maturity in soybean. As an algorithm for analysis, we took “Simple Tidy GeneCoEx” (Li C. et al., 2023), which was largely chosen due to the simplicity of its use. As a result, we reduced the initial list of candidate genes by almost three times. However, this approach has limitations, since the casual gene does not need to be co-expressed with the bait genes or be associated with the response of soybeans to photoperiod, for example, temperature is also an important factor affecting soybean flowering (Yang et al., 2022; Wu et al., 2023; Yao and Zhang, 2024). Nevertheless, co-expression analysis allowed for a more comprehensive prioritization of candidate genes and the identification of interesting genes worthy of further study (**Supplementary Table S4**).

Much more loci are found in the conditions of the Novosibirsk region (**Supplementary Figure S2**, **Table 1**). Apparently, this is due to differences in environmental conditions, in particular, a stronger residual response of soybeans to photoperiod in Novosibirsk. Two loci qFM-23 and qFM-27 are found in both geographical regions. Interestingly, in Novosibirsk they are associated with flowering, and in Orel with the maturity of soybean (**Supplementary Figure S2**, **Table 1**). The qFM-23 locus co-localizes with the previously discovered “Reproductive stage length 4-g3” locus (Copley et al., 2018), and qFM-27 does with the previously discovered “qMG-16.4” (Zimmer et al., 2021) (**Table 1**). Interestingly, Copley et al. in the first work also used early maturing germplasm collections (Copley et al., 2018). Perhaps this locus is important and provides variation only on an early maturing genetic background.

For the qFM-23 loci, we propose *Glyma.13G177400* as the most likely candidate gene. *Glyma.13G177400* codes *GmSPL3c* (*SQUAMOSA PROMOTER BINDING PROTEIN LIKE 3*), which in Arabidopsis directly regulates *FLOWERING LOCUS T* (*FT*) expression through the miR156-SPL module (Jung et al., 2016). Cao et al. showed that a similar module, miR156-SPL, is present in the soybean flowering pathway (Cao et al., 2015b). For *GmSPL3c*, we found a negative correlation with the *E3* gene (**Supplementary Table S4**), which is consistent with the fact that GmmiR156 is down-regulated by the *E1*-*E4* genes (Cao et al., 2015b). In the SoyOmics database, we found two non-synonymous substitutions in *GmSPL3c* that form three haplotypes (**Supplementary Table S5**). The first and third haplotypes mature significantly later compared to the second (**Figure 5D**). Additionally, early maturing haplotypes hap1 and hap3 are more common in Chinese cultivars from northern latitudes (**Figure 5B**). Additionally, hap2 is found only in cultivated soybeans, with its frequency increasing from landraces to improved cultivars (**Supplementary Table S5**). Interestingly, the soy19413364 (C>A) mutation is located in the SBP domain and leads to the replacement of histidine (positively charged) with glutamine (neutrally charged), which may somehow affect DNA binding or recognition of cis elements. In our collection, we did not detect these two polymorphisms using dCAPS markers (**Supplementary Table S6**); all accessions carry hap1. However, we identified another mutation, Soy130029141524SNP, using a CAPS marker (**Figure 8C**). The LD between the high p-value SNPs from GWAS results for Novosibirsk is higher than the mean (r² = 0.56, D’ = 0.86), while for Orel, it is below the mean (r² = 0.39, D’ = 0.68). The mutation shows an association with BLUP_DTF and BLUP_DTM in the Novosibirsk and Orel regions (**Figure 8D**). The C allele, associated with early flowering and maturity, is predominant in accessions from Western Siberia (**Figure 8E**). The 5’ UTR of *Glyma.13G177400* contains multiple start codons, and Soy130029141524SNP forms an additional start codon in the 5’ UTR with an out-of-frame overlapping upstream ORF (uORF) (**Figure 8A**). Such types of uORFs have the most repressive effects on mORF translation (Wang J. et al., 2024). It is also interesting that the uORF formed by the Soy130029141524 SNP contains a poly-A sequence (**Figure 8B**). In dicots, AT-rich (Nakagawa et al., 2007) and AAAAAAA(A/C)AAUGGCU (Joshi et al., 1997) sequences are indicated as preferred nucleotides in the Kozak sequence. In Arabidopsis. an A residue in positions from −1 to −5 is associated with a high-level translational efficiency (Kim et al., 2014). The insertion of the trinucleotide AAA before the start-codon increases a translation efficiency of *OsSBI* (*Oryza sativa shortened basal internodes*) and reduces the plant height (Wang H. et al., 2024). Thus, we hypothesize that the uORF formed by the G allele of Soy130029141524SNP has a higher translation efficiency than the mORF, leading to a loss of function of *GmSPL3c*, and as a result, to late flowering and maturity.

For the qFM-27 loci, we propose *Glyma.16G206400* as the most likely candidate gene. *Glyma.16G206400* codes *PMI1* (*PLASTID MOVEMENT IMPAIRED1)*, a gene involved in blue-light-induced chloroplast movement and abscisic acid (ABA) signaling pathway (Rojas-Pierce et al., 2014). ABA may control plant flowering under osmotic stress (Martignago et al., 2020). Expression of *Glyma.16G206400* correlates positively with *Tof16* and negatively with *GmLUX1* and *GmLUX2* (**Supplementary Table S4**). We cannot assume how this gene can regulate flowering at the molecular level. *Glyma.16G206400* has the most non-synonymous substitutions. Among the candidate genes studied, 7 mutations were found that form 6 main haplotypes (**Supplementary Table S5**). The early maturing haplotypes, hap1 and hap2, are significantly different from the late maturing haplotypes, hap4 and hap5 (**Figure 5D**). No pattern was found in the distribution of haplotypes across latitudes in China (**Figure 5B**). The late-maturing haplotype, hap4, was found only in cultivated soybean (**Supplementary Table S5**).

Of the loci that were found only in single regions, we can highlight: qDTF-7, qDTF-13, and qDTM-18, since they carry the most interesting candidate genes.

For locus qDTF-7, we propose *Glyma.03G181400* and *Glyma.03G177500* as the most likely candidate genes. The first gene, *Glyma.03G181400*, codes *EIN2* (*ETHYLENE-INSENSITIVE 2*) homologs (Robison et al., 2019), a gene that participates in signal transmission from ethylene. Treating soybeans with ethylene inhibitors accelerates flowering (Cheng et al., 2013), and knockout of *ETHYLENE INSENSITIVE-LIKE* genes in soybean leads to early flowering (Cheng et al., 2023). Two non-synonymous substitutions were found in *Glyma.03G181400* (**Supplementary Table S4**), but they were not associated with BLUP_BBD and BLUP_MD. Second gene, *Glyma.03G177500* codes *AP2-like factor, euAP2 lineage (AP2)* and is an ortholog of the *TOE1* (*TARGET OF EARLY ACTIVATION TAGGED 1*) gene, which in Arabidopsis binds the *FT* promoter near the CO-binding site to inhibit *CO* activity (Zhang B. et al., 2015). In soybean, Li et al. cloned *TOE4b*, which belongs to the AP2/ERF family and reveled that *TOE4b* represses flowering time by binding to the *GmFT2a* and *GmFT5a* promoters (Li H. et al., 2023). Interestingly, the expression of *Glyma.03G177500* is negatively correlated with *GmFT2a* and possibly has a similar molecular function as *TOE4b* (**Supplementary Table S4**). Wang et al. showed that the *toe1* mutant can be partially restored by the expression of *Glyma.03G177500* in Arabidopsis (Wang T. et al., 2016). Three mutations were found in *Glyma.03G177500*, which form four main haplotypes (**Supplementary Table S5**). For BLUP_BBD, the early flowering haplotypes, hap1 and hap3, are significantly different from the late flowering haplotype, hap2 (**Figure 5C**). A similar situation is observed in BLUP_MD, where the early maturing haplotypes, hap1 and hap3, are significantly different from the late maturing haplotypes, hap2 and hap4 (**Figure 5D**). Haplotypes hap1 and hap3 are predominant in Chinese cultivars from northern latitudes (**Figure 5B**). Additionally, the late maturing haplotype, hap2, is completely predominant in wild soybeans, while the frequencies of early maturing haplotypes, hap1 and hap3, increase from landraces to improved cultivars (**Supplementary Table S5**). It’s possible that these early maturing haplotypes were selected during the process of domestication and secondary improvement of soybean. Haplotypes hap1 and hap3 differ from hap2 and hap4 by the substitution soy4989324 (T>C). The earliest maturing hap3 differs from other haplotypes by the substitution soy4989323 (G>A) (**Figure 5D**, **Supplementary Table S5**). These two non-synonymous mutations are of interest for further study. Both mutations are located outside of both AP2 (124-183 a.c. and 216-260 a.c.) domains of the protein; perhaps they somehow affect the 3D structure of the protein or reduce the binding of *Glyma.03G177500* to blue light receptors, cryptochromes (CRYs). In Arabidopis, Du et al. showed that CRY1 and CRY2 physically interact with TOE1 and TOE2 and the entire N-terminal domain of TOE1 (amino acids 1–292) and TOE2 (amino acids 1–325) comprising both AP2 domains are essential for interaction with CRY2 (Du et al., 2020). The soy4989324 mutation is present in our collection and shows a weak association with BLUP_DTF and BLUP_DTM in Novosibirsk and a stronger association with BLUP_DFT in Orel (**Figure 6C**). The LD between the high p-value SNP and soy4989324 is below the mean (r^2^ = 0.25, D` = 0.59). Interestingly, the early flowering and maturing allele (C) is also dominant in accessions from Western Siberia (**Figure 6B**), as well as in Chinese cultivars from northern latitudes (**Figure 5B**).

For locus qDTF-13, we propose *Glyma.06G213100* as the most likely candidate gene. The genomic region containing qDTF-13 has previously been reported to be associated with soybean flowering time (**Table 1**). *Glyma.06G213100* codes DELLA protein, DELLA proteins are the core elements in gibberellins (GAs) signal transduction pathway (Anwar et al., 2021). Also DELLA proteins provide a link between GA signaling pathway and photoperiod depended flowering through the DELLA-CO interaction, which represses the transcriptional function of the CO protein (Wang H. et al., 2016). DELLA proteins are a negative regulator of flowering, and GA-dependent degradation of DELLA proteins promotes the expression of *FT* and *SOC1* (*SUPPRESSOR OF OVEREXPRESSION OF CONSTANS 1*) genes in Arabidopsis (Fukazawa et al., 2021). Interestingly, the expression of *Glyma.06G213100* has a negative correlation with *GmFT2a* and *GmFT5a* and a positive correlation with the evening complex genes *GmEL3* and *GmLUX2* (**Supplementary Table S4**). This is consistent with the results of Fukazawa et al., who showed the involvement of *ELF3* and *LUX* in GA-dependent flowering in Arabidopsis (Fukazawa et al., 2021). It is possible that GA controls flowering in soybean at the molecular level in a similar way, but this requires further study. In *Glyma.06G213100*, we found only one mutation, soy8754921 (A>T), resulting in two haplotypes. Cultivars carrying the haplotype hap2 flower and mature later than those with hap1 (**Figures 5D, C**). Recently, He et al. have been studied the soybean DELLA genes and found an association between *Glyma.06G213100* haplotypes and soybean flowering in the middle and high latitude of China (He et al., 2022). Also, the frequency of hap2 in China cultivars increases from south to north latitudes (**Figure 5B**). However, the frequency of haplotypes does not significantly change from wild to cultivated soybean (**Supplementary Table S5**). The soy8754921 mutation is present in our collection and is strongly linked to the high p-value SNP (r² = 0.85, D` = 0.97). This SNP shows a strong association with BLUP_DTF in both regions and with BLUP_DTM in Novosibirsk (**Figure 7B**). The early maturing allele (T) is predominant in accessions from Western Siberia (**Figure 7D**). It is important to note that *Glyma.06G213100* is located relatively close to the main flowering regulator gene *E1*, approximately 1.2 Mb away. Moreover, soy8754921 shows above average LD with the *E1* alleles (r^2^ = 0.58, D` = 0.82) Therefore, we additionally checked the association of soy8754921 with BLUP_DTF, taking into account the genetic background of *E1*. Although there are few recombinant genotypes, they significantly differ from each other (**Figure 7C**) in BLUP_DTF in both regions.

For the qDTM-18 loci, we propose *Glyma.08G255200* as the most likely candidate gene, which codes *GmCOL1b* (*CONSTANS Like 1b*). In Arabidopsis, *CONSTANS* (*CO*) is a key gene that integrates clock and light signals to provide photoperiod-specific expression of *FT* (Song YH. et al., 2013). In Soybean *GmCOL1b* acts as flowering repressor under LD conditions (Wu et al., 2014; Cao et al., 2015a). However, the association between natural variation in *GmCOL1b* and flowering/maturity time has not yet been reported. Moreover, Awal Khan et al. showed very high conservation of the *GmCOL1b* in soybean cultivars (Awal Khan et al., 2022). We also found that *GmCOL1b* is highly conserved in improved soybean cultivars, according to data from SoyOmics (**Supplementary Table S3**). Nevertheless, this candidate gene is of interest for further study and it is possible that our population contains polymorphisms that affect its function. We also do not exclude that this locus is false positive due to the relatively low frequency of the minor allele (MAF = 0.093).

## 5 Conclusion

Among the proposed candidate genes, we found haplotypes associated with soybean flowering and maturity in *Glyma.03G177500*, *Glyma.06G213100*, and *Glyma.13G177400*, and developed DNA markers to identify these haplotypes. Early flowering and maturing haplotypes of these genes are predominant in accessions from northern regions of China and Western Siberia, potentially providing soybean adaptation to northern latitudes. The loci and candidate genes identified in this study may serve as a valuable resource for soybean breeding to fine tune soybean flowering and maturity time. Validation of these loci in other soybean populations or hybrid progeny will be our next goal.

## Data Availability

The SNPs data presented in the study are deposited in the Zenodo repository, https://doi.org/10.5281/zenodo.13879245. Phenotyping data used in this study is available in **Supplementary Material**.
