# Supplementary figures and images for: Genome-wide association study revealed some new candidate genes associated with flowering and maturity time of soybean in Central and West Siberian regions of Russia
Source: Front Plant Sci. 2024 Oct 11;15:1463121. doi: 10.3389/fpls.2024.1463121 (PMC11502416; doi:10.3389/fpls.2024.1463121)

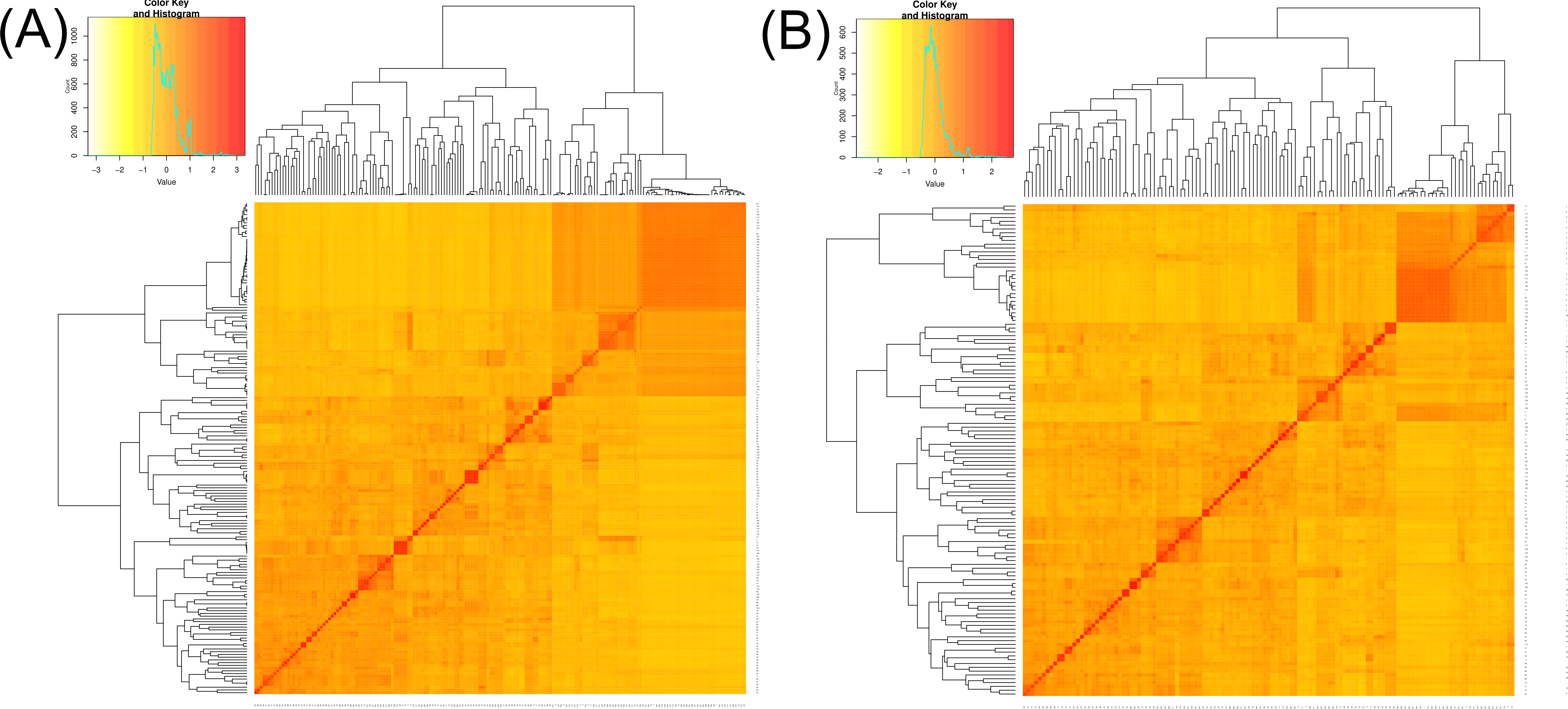

Supplement: Supplementary file 1 [file Image1.jpeg]

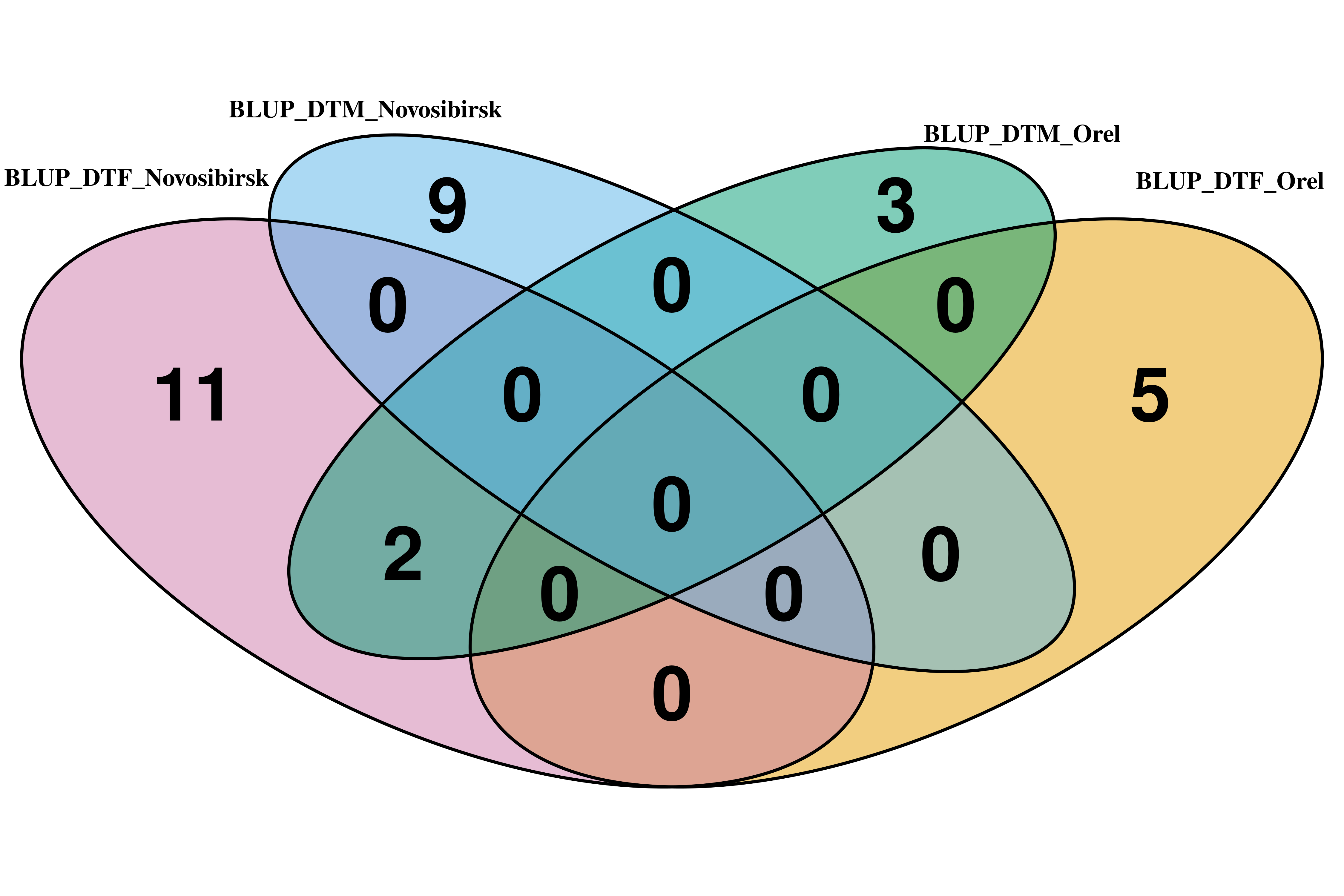

Supplement: Supplementary file 2 [file Image2.jpeg]

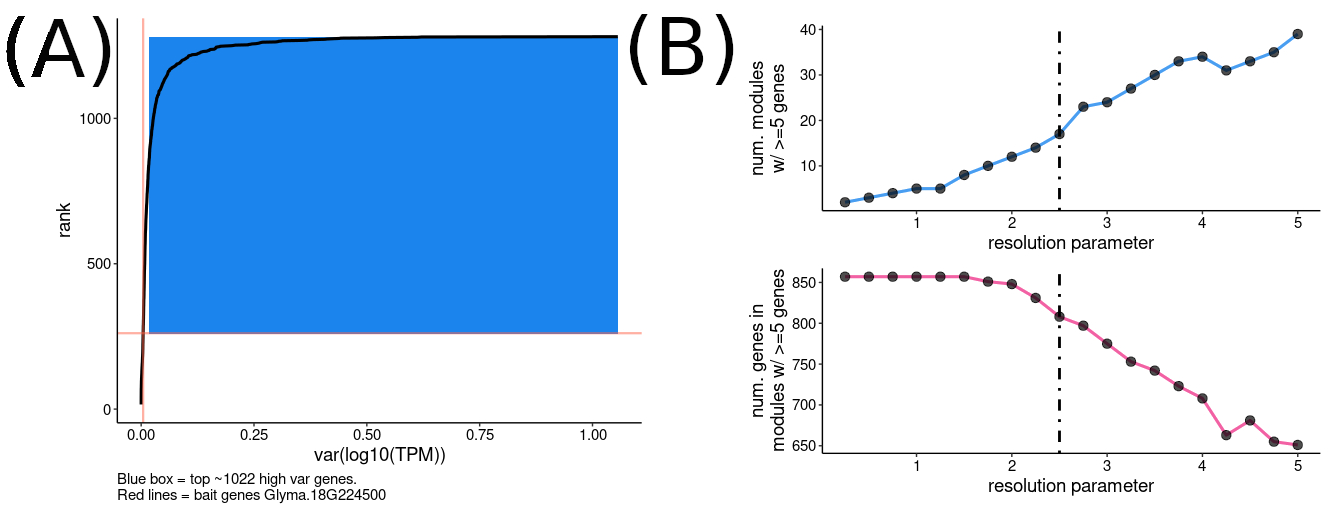

Supplement: Supplementary file 3 [file Image3.jpeg]
